# The impact of uncertainty estimation on radiomic segmentation reproducibility and scan–rescan repeatability in kidney MRI

**DOI:** 10.1002/mp.17995

**Published:** 2025-07-15

**Authors:** Rossella Damiano, Ettore Lanzarone, Francesca Lussana, Giulia Villa, Alexander J Daniel, Susan Francis, Anna Caroli, Elisa Scalco

**Affiliations:** ^1^ Institute of Biomedical Technologies (ITB) National Research Council (CNR) Milan Italy; ^2^ Department of Management Information and Production Engineering University of Bergamo Bergamo Italy; ^3^ Department of Bioengineering Istituto di Ricerche Farmacologiche Mario Negri IRCCS Bergamo Italy; ^4^ Sir Peter Mansfield Imaging Centre University Park University of Nottingham Nottingham UK; ^5^ NIHR Nottingham Biomedical Research Centre Nottingham UK

**Keywords:** MRI kidney segmentation, radiomic stability, U‐Net, uncertainty quantification

## Abstract

**Background:**

Radiomics holds great potential but is hindered by segmentation and scan–rescan variability, which affect the reproducibility and repeatability of radiomic analysis, respectively. Recently, deep learning (DL) has shown promise in improving segmentation accuracy, thereby enhancing radiomic stability. Moreover, including uncertainty quantification into DL models could provide confidence assessments for segmentations, ultimately improving the trustworthiness and robustness of radiomic outputs.

**Purpose:**

This study investigated whether the reproducibility and repeatability of radiomic features, in relation to segmentation and scan–rescan variability, respectively, could be enhanced by extracting features exclusively from confidently segmented regions, rather than from regions defined without accounting for uncertainty‐related information. Additionally, this study assessed whether stable features derived from uncertainty‐informed segmentation could improve the classification of healthy versus pathological subjects.

**Methods:**

A publicly available kidney MRI dataset, including subjects with chronic kidney disease (CKD) and healthy controls (HC), was used to assess the robustness of the segmentation methods across diverse clinical scenarios. A deterministic U‐Net model was first implemented to generate kidney masks without considering segmentation uncertainty. Then, Monte Carlo dropout (MCD) and test‐time augmentation (TTA) were applied to address uncertainty in DL‐based segmentation. Both methods were trained using the traditional Dice loss and a recently proposed Dice Plus loss to improve model calibration. Confidence level‐based masks were defined from the predictions with uncertainty, identifying kidney regions segmented with different levels of certainty. Radiomic features were extracted from ground truth masks, deterministic masks, and confidence level‐based masks. These features were grouped into four classes based on their intraclass correlation coefficient values in relation to both segmentation and scan–rescan variability. Finally, based on the identified stable features, a classification model was developed for each approach to distinguish between CKD and HC subjects.

**Results:**

The accuracy results were comparable across all the implemented models, with Dice score coefficients consistently above or near 0.9. Most radiomic features were unstable with respect to both segmentation and scan–rescan variability when uncertainty information was not considered. However, including uncertainty increased the number of features repeatable with respect to scan–rescan variability in both CKD and HC subjects. The greatest improvement was observed with the MCD approach trained with the Dice Plus loss, whereby the number of repeatable features increased from 24 to 70 out of 105 in total, for both CKD and HC subjects. Improvements in reproducibility with respect to segmentation variability were not consistent across methods and subject groups. Regarding the classification analysis, all uncertainty‐based approaches performed comparable to the references in terms of ROC curves.

**Conclusions:**

Integrating uncertainty quantification into DL‐based segmentation for radiomic features extraction represents a promising approach to enhance the robustness of radiomic analysis against segmentation and scan–rescan variability, as well as its ability in distinguishing pathological from healthy subjects. Additionally, such integration improves the reliability and interpretability of radiomic analysis, contributing to more informed clinical decision‐making.

## 1 INTRODUCTION

In recent years, deep learning (DL) has emerged as a pivotal tool in medical imaging, achieving remarkable success in various tasks, particularly in medical image segmentation.^1^ DL can employ convolutional neural networks (CNNs) with multiple layers to extract and analyze complex features from images, thereby enabling the detection of intricate patterns that may be challenging for humans to discern.^2^ This approach allows for highly precise and efficient identification and delineation of anatomical structures, frequently outperforming traditional segmentation methods.^3^ Moreover, DL techniques have yielded promising results even when applied to relatively small datasets, which is especially advantageous in medical imaging due to the often limited availability of data.^4^, ^5^

However, the adoption of DL models in clinical practice is hindered by the reliability of their outcomes.^6^ The inherent lack of transparency due to their black‐box nature limits DL models' clinical applicability. In fact, understanding the rationale behind a model's predictions could enhance confidence and accelerate acceptance by medical professionals. Another critical challenge is the tendency of DL models to produce miscalibrated predictions. Ideally, a well‐calibrated model should generate class probabilities that accurately reflect the true likelihood of an event. However, DL models are often miscalibrated by being overconfident in their misclassifications, which can impact their reliability in clinical applications.^7^ Furthermore, the accuracy and certainty of automatic delineation of the regions of interest (ROIs) can be affected by the quality of the manual segmentation used as a reference during training. Traditional DL models fail to provide an accurate quantification of confidence regarding their output, since conventional quality metrics offer only a general assessment of model performance without addressing predictions' uncertainty.

The integration of tools that evaluate segmentation uncertainty in DL models can be pivotal for enhancing trustworthiness, especially in a critical domain, such as healthcare.^8^, ^9^ Different methods have been proposed to quantify uncertainty in DL algorithms.^10^, ^11^ Bayesian CNNs (BCNNs), which offer predictions in terms of their posterior probability density,^12^ are widely used for this purpose. More specifically, at inference time, BCNNs provide probability density functions for the output label probabilities in each voxel, rather than the single point value traditionally estimated by deterministic CNNs. In this way, it is possible to extract the mean prediction and its variability, to estimate the model's predictive uncertainty. A popular approach within Bayesian inference is the Monte Carlo dropout (MCD),^13^ which integrates dropout layers into CNNs, converting a deterministic CNN into a BCNN with faster training times compared to other variational inference techniques. More precisely, MCD provides several outputs from the same trained network, repeating the inference with dropped weights, which are taken as samples to build the posterior probability density function of the prediction voxelwise. Non‐Bayesian methods have also been developed to address uncertainty quantification. Among them, test‐time augmentation (TTA) provides different predictions through different augmentations of the input data, which are then combined to improve robustness and quantify uncertainty.^14^

Recently, in the context of conventional radiomics,^15^ here intended as the extraction of handcrafted features from medical images, DL achieved interesting results when introduced in the image processing steps of its pipeline.^16^ Radiomics faces stability challenges due to the variability and uncertainty present at each stage of its pipeline,^17^ which hinder its potential in building robust and generalizable predictive models.^18^ In this context, stability refers to the ability of radiomic features to remain consistent despite these sources of variability, which arise from inconsistencies in the delineation of the regions of interest and from changes in image data, caused by factors such as patient positioning. Specifically, segmentation and scan–rescan variabilities have been reported as major factors affecting radiomic reliability.^19^ The use of standard DL architectures for automatic image segmentation has shown potential to improve the stability of radiomic features.^16^

The application of radiomics to the renal MRI is of high interest, as documented by recent studies focusing on chronic kidney disease (CKD).^20^, ^21^ In fact, radiomic features derived from renal MRI have proven effective in differentiating between healthy individuals and those with mild to moderate CKD, with a sensitivity of 93%, a specificity of 70%, and an area under the curve (AUC) of 0.95. Radiomics has also shown promise in developing predictive models for CKD progression, with four radiomic features able to classify participants as rapid versus non‐rapid CKD progressors, showing a sensitivity of 71%, a specificity of 43%, and an AUC of 0.75.^22^ However, radiomics stability in this context has so far been little studied, and effective strategies to cope with segmentation and scan–rescan variability are still lacking.

In this study, we aim to evaluate whether integrating DL segmentation uncertainty into the radiomics workflow can effectively address the challenges posed by segmentation and scan–rescan variability that impact the reliability of radiomic analysis. Our primary objective is to investigate whether limiting the extraction of radiomic features to regions with high‐confidence segmentation enhances stability. Additionally, we compare various approaches for uncertainty quantification in CNNs, employing both traditional and innovative loss functions to improve model calibration. Finally, we assess the impact of this approach on the capability to discriminate healthy and pathological subjects. We apply the analysis to a publicly available renal MRI dataset to address the specific unmet clinical needs in the previously cited renal field; however, the results can be extended to other contexts as well as imaging modalities beyond MRI.

By including an uncertainty assessment step into the DL‐based segmentation of the radiomics pipeline, we propose an automated method to enhance the reliability and interpretability of model outcomes, ultimately facilitating the application of radiomics in clinical practice.

## 2 METHODS

### 2.1 MRI dataset

The dataset used in this study included T2‐weighted kidney MRI coronal scans of 60 subjects obtained from a public repository.^23^ Thirty subjects had CKD, while thirty were healthy controls (HCs). Ten subjects (five HC and five CKD) were scanned five times in a single scanning session, with repositioning between each acquisition. The images were acquired by a 3T Philips Ingenia system (Philips Medical Systems, Best, the Netherlands) using a 2D T2‐weighted half‐Fourier single‐shot turbo spin echo sequence optimized to achieve the maximum contrast between the kidneys and surrounding tissue.^24^

For each scan, the corresponding kidneys' masks were manually segmented using the free open‐source software MRIcron. These manual segmentations, outlined by one of three trained observers with an average of two years of experience in kidney segmentation, were used as the ground truth (GT) for the segmentation task.

The dataset was split into training and test sets, ensuring that the division was stratified by patient. Specifically, all the slices from the 50 subjects (25 with CKD and 25 HC) with a single acquisition were used to form the training set, comprising a total of 649 2D MRI slices, which was further randomly divided into training and validation sets with an 80%–20% split, without stratifying by patient. On the other hand, all the slices from the 10 subjects (5 CKD and 5 HC) scanned 5 times with repositioning were exclusively allocated to the test set, totaling 650 2D MRI slices. These scans were used to both test the proposed segmentation algorithms and develop the subsequent radiomic analysis.

### 2.2 CNN architectures for segmentation

We adopted the deterministic U‐Net architecture^25^ for kidney segmentation following the original implementation developed for the image dataset,^24^ whose structure is provided in Figure S1.

Starting from this deterministic U‐Net model, we introduced uncertainty quantification by considering Bayesian and non‐Bayesian approaches. These allow multiple predictions to be generated from the same input, thereby enabling uncertainty quantification. More specifically, we considered the following:

- **Monte Carlo dropout**: This is a Bayesian approach in which dropout layers are inserted into the network after the final convolution or deconvolution operation within each encoder or decoder unit, respectively, and are kept active during both training and inference phases.^13^ During inference, the network executes T stochastic forward passes, where each pass involves the random deactivation of a specified percentage of input neurons (i.e., the dropout rate). Each stochastic forward pass produces a prediction that can be interpreted as a sample from a slightly varied network configuration. This process yields a distribution of outcomes, providing a probabilistic view of model predictions rather than a single deterministic output. The architecture of the Bayesian 2D U‐Net used for MCD is illustrated in Figure S2.
- **Test time augmentation**: This is a non‐Bayesian approach that involves applying T−1 different data augmentation transformations (e.g., rotations, translations, and flips) to the input test image.^14^ The deterministic U‐Net model makes predictions on the original input image and on the T−1 augmented images; then, these T predictions are used to assess uncertainty.

### 2.3 CNN training

Data preprocessing was applied to prepare the data for training the CNN architectures following the same workflow adopted by Daniel et al. in the study providing the image data.^24^

First, voxel intensities were bounded between the mean voxel intensity minus half the standard deviation (SD) of the volume and the mean voxel intensity plus four times the SD of the volume. Then, they were rescaled between 0 and 255. Each volume was then divided into 2D coronal slices and resized to a 256×256 pixel matrix. As mentioned above, 20% of the training set was set aside for validation, with randomized slice order within both the training and the validation sets.

Data augmentation was implemented during training to enhance variability, thereby mitigating the risk of overfitting and improving model performance and generalizability on unseen data.^26^ This augmentation process involved applying random transformations to the training images and their corresponding masks, including horizontal and vertical shifts (up to 25%), zoom adjustments (ranging from 0.75 to 1.25 times), rotations (up to 20 degrees), and shearing (up to 5 degrees).

The deterministic and the Bayesian MCD networks were trained using the Adam optimizer^27^ and two loss functions:

1. Dice score loss function ($L_{\text{DSC}}$), based on the classical Dice score coefficient (DSC) and defined as follows (adapted from Yeung et al.^28^):
(1) $$L_{\text{DSC}}=1-\text{DSC}=1-\frac{2\sum_{i=1}^{N}p_{i}y_{i}}{\sum_{i=1}^{N}p_{i}+\sum_{i=1}^{N}y_{i}},$$
  where $y_{i}$ and $p_{i}$ are the GT and the prediction for pixel i, respectively.
2. Dice Plus loss function ($L_{\text{DSC++}}$), introduced by Yeung et al.^28^ to improve model calibration, which ensures accurate estimation of predictive uncertainty.^29^ $L_{\text{DSC++}}$ exponentially penalizes overconfident (i.e., predictions for which the model assigns a probability to a given class that does not match the true likelihood) false positive and false negative predictions by introducing a focal parameter γ as follows:
(2) $$L_{\text{DSC++}}=1-\frac{2\sum_{i=1}^{N}p_{i}y_{i}}{2\sum_{i=1}^{N}p_{i}y_{i}+\sum_{i=1}^{N}{\left[p_{i}\left(1-y_{i}\right)\right]}^{\gamma }+\sum_{i=1}^{N}{\left[\left(1-p_{i}\right)y_{i}\right]}^{\gamma }}.$$

Summing up, four stochastic networks were considered, denoted by the name of the stochastic approach (MCD or TTA), followed or not by the label PLUS when trained using $L_{\text{DSC++}}$ or $L_{\text{DSC}}$, respectively: MCD, TTA, ${\text{MCD}}_{\text{PLUS}}$ and ${\text{TTA}}_{\text{PLUS}}$.

Moreover, the deterministic U‐Net trained using the $L_{\text{DSC}}$ was considered as the reference model.

Hyperparameter tuning was performed, and the optimal configuration was chosen to minimize the loss on the validation set. The resulting optimized parameters used for training the networks were a learning rate of $10^{-4}$, 100 epochs, a batch size of 16, and a dropout rate of 0.25.

All CNNs were implemented using TensorFlow (version 2.14) in Python (version 3.11.8), and the training was carried out on a Microsoft Windows machine equipped with an Intel Core i7‐13700 2.10 GHz CPU, 32 GB of installed RAM, and a NVIDIA GeForce RTX 4070 Ti GPU. The code is publicly available at https://zenodo.org/records/15188948.

### 2.4 CNN inference and uncertainty quantification

The number of predictions T obtained from each test image was set to 50 for both the MCD and TTA approaches. This number was large enough to ensure stable and reliable uncertainty estimates, that is, the coefficients of variation of the extracted features did not vary by increasing this number further.

In particular, TTA predictions were generated by applying data augmentation during inference. Each input image was augmented 49 times (i.e., T−1) using flipping, horizontal and vertical shifts, rotations, and combinations of these.

The predicted mask ${\bar{p}}_{i}$ in pixel i (i=1,⋯,N) was computed as the average of the estimated probabilities $p_{it}$ in the pixel across the T samplings:^30^

(3) $${\bar{p}}_{i}=\frac{1}{T}\sum_{t=1}^{T}p_{it}.$$

Then, ${\bar{p}}_{i}$ was binarized pixel‐wise to generate mean prediction (MP) masks.

The predictive uncertainty in pixel i was evaluated by considering the contributions of both aleatoric uncertainty, stemming from the variability inherent in the data, and epistemic uncertainty, which arises from limited knowledge or information of the underlying data distribution^31^, ^32^ These are determined as follows:^30^, ^33^, ^34^

(4) $$\begin{array}{ccc} U_{i}^{\left(\text{pred}\right)} & = & U_{i}^{\left(\text{aleat}\right)}+U_{i}^{\left(\text{epist}\right)}=\frac{1}{T}\sum_{t=1}^{T}p_{it}\left(1-p_{it}\right)\\ & & +\;\frac{1}{T}\sum_{t=1}^{T}{\left(p_{it}-{\bar{p}}_{i}\right)}^{2}. \end{array}$$

### 2.5 Radiomic analysis

#### 2.5.1 Mask identification

The initial step in radiomic analysis is the definition of the masks (ROIs) from which features are to be extracted, which is critical for radiomics reproducibility and repeatability.

A single prediction mask was obtained from the deterministic network, referred to as *deterministic prediction* (DP), which did not include uncertainty‐related information. Similarly, the GT, provided in the dataset, did not include information related to uncertainty.

In addition, T=50 predictions were generated for each method that accounted for uncertainty, as described in Section 2.4. In these cases, confidence level ${\mathrm{CL}}_{th}$ masks, with segmentation confidence level th ranging from 10 to 100 with a step size of 10, were considered to evaluate the impact of integrating segmentation uncertainty on radiomics robustness.

${\mathrm{CL}}_{th}$ masks were extracted voxelwise from the T=50 predicted masks by including a voxel if it was classified as part of the kidney region in at least th% of the T replications. For instance, ${\mathrm{CL}}_{10}$ denotes the region of voxels classified as kidney by at least 10% of the 50 replications, while ${\mathrm{CL}}_{100}$ represents the region of voxels classified as kidney by all 50 segmentations.

**FIGURE 1:**
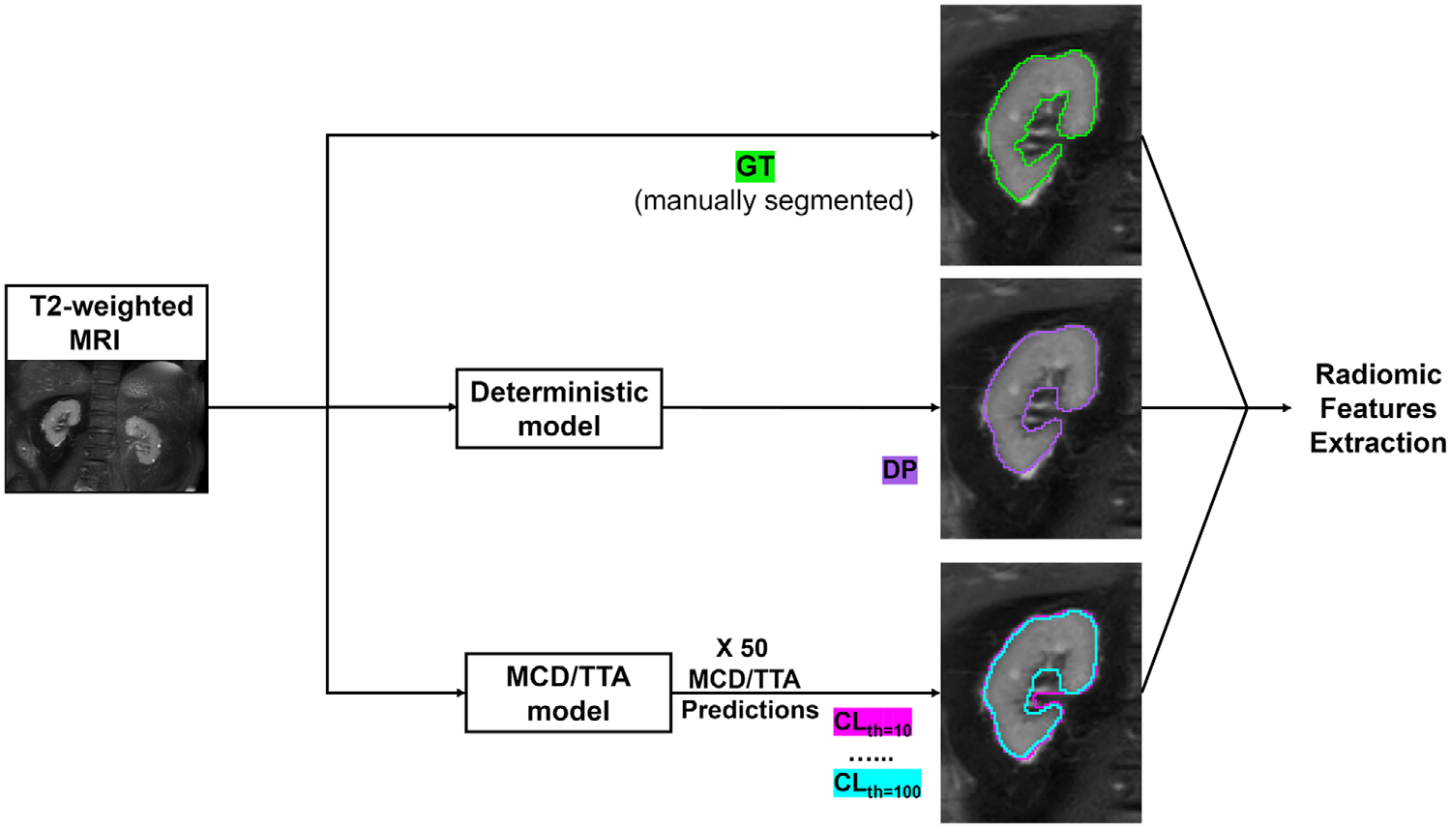
Description of the workflow used to define masks for feature extraction.

This workflow is outlined in Figure 1.

#### 2.5.2 Feature extraction

Radiomic features were extracted from the GT, DP, and ${\mathrm{CL}}_{th}$ masks using the open‐source package Pyradiomics^35^ in Python.

A total of 105 radiomic features were computed, in accordance with the imaging biomarker standardization initiative (IBSI) guidelines.^36^ These included first‐order statistics, shape‐based features, and textural features derived from various textural matrices, such as the gray‐level co‐occurrence matrix (GLCM), the gray‐level run length matrix (GLRLM), the gray‐level size zone matrix (GLSZM), the gray‐level dependence matrix (GLDM), and the neighboring gray tone difference matrix (NGTDM).

A common resolution of [1.458,1.458,5.5]mm, which corresponds to the most common acquisition voxel size, was considered to resample the test images, using the BSpline interpolator. *Z*‐score intensity standardization was then performed, and a fixed number of bins (64) was set for intensity discretization.

Due to the relevant difference in voxel size between the third dimension and the in‐plane resolution, all features were calculated slice by slice from the original test acquisitions without applying any filter, and these calculations were then averaged.^37^

### 2.6 Evaluation metrics

#### 2.6.1 Segmentation accuracy and uncertainty metrics

The performance of DL segmentation models was assessed in terms of accuracy and uncertainty.

The accuracy was evaluated using the DSC,^38^ which measures the overlap between the DP mask and the GT for the deterministic model, and between the MP mask and the GT for the models accounting for uncertainty, as introduced in Section 2.4. This metric was computed on the entire MRI volumes ($DSC_{\text{3D}}$) of the test set. Higher DSC_3D_ values indicate a greater degree of overlap—that is, better segmentation performance.

The uncertainty was assessed using the Kullback–Leibler (KL) divergence^39^ between the distribution of predictive uncertainties for correctly classified voxels and that for incorrectly classified voxels, with these probability density functions approximated with the histogram of uncertainty values. Higher KL divergence values suggest that the model effectively distinguishes between correct and incorrect predictions, with the last ones expected to be associated with higher uncertainty values, thus indicating better performance in terms of uncertainty estimation.

#### 2.6.2 Stability analysis

Radiomic stability was analyzed in terms of both reproducibility with respect to segmentation variability and repeatability across scan–rescan acquisitions, and it was assessed using the intraclass correlation coefficient (ICC),^40^, ^41^ a reliability index based on the analysis of variance.

Reproducibility with respect to segmentation variability was estimated by ICC (for two‐way mixed effects, consistency, and multiple raters/measurements^41^) between manually and automatically delineated kidney regions ($ICC_{S}$). Repeatability with respect to variability in scan–rescan acquisitions was estimated by the ICC (for two‐way random effects, absolute agreement, and multiple raters/measurements^41^) among the five scan repetitions per subject on the kidney mask ($ICC_{A}$), considering CKD and HC subjects together to ensure enough data for analysis, with the hypothesis that scan–rescan variability is not dependent on the subject group. Statistical tests were performed on the distributions of $ICC_{S}$ and $ICC_{A}$ for each method, compared to the approaches without confidence information, to evaluate statistical differences between deterministic and uncertainty‐informed stability analysis. Specifically, a two‐tailed Wilcoxon signed‐rank test with a significance level of 0.05 was used.

**FIGURE 2:**
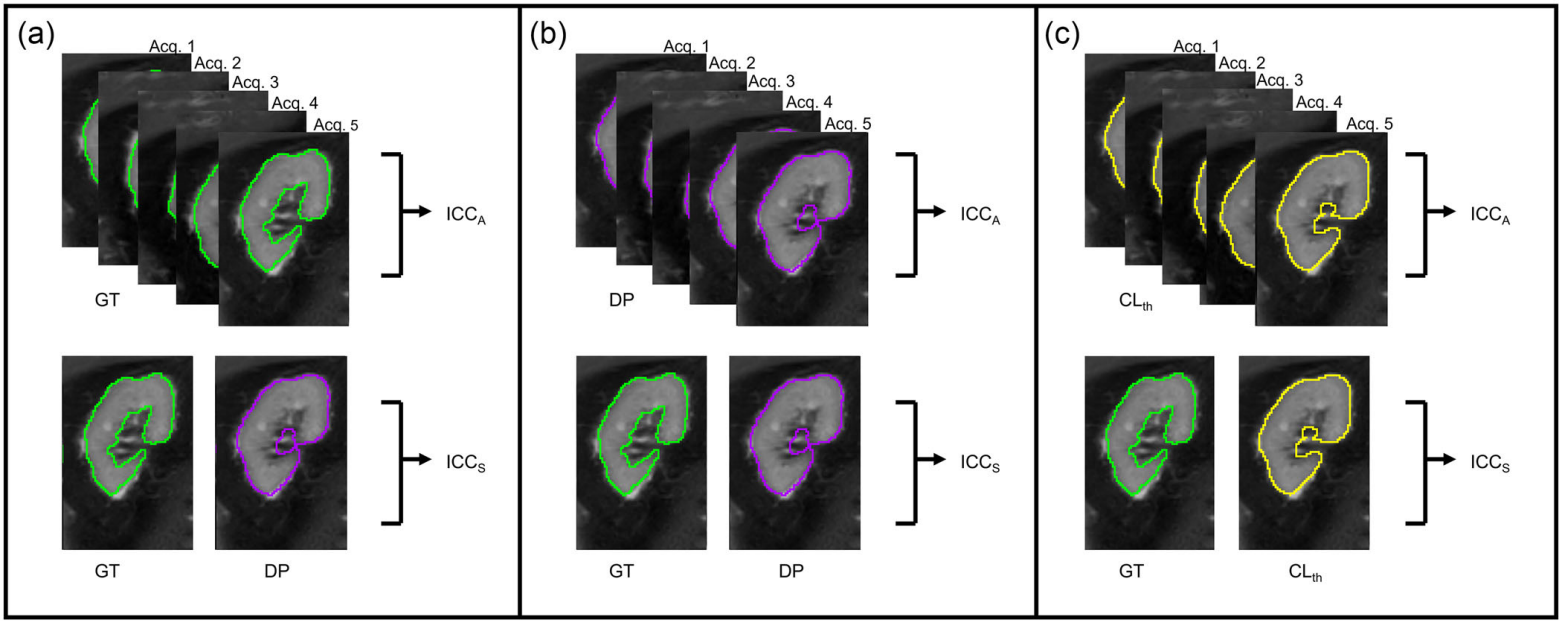
Graphical description of the reproducibility and repeatability analyses for ICC computation: (a) manual GT approach; (b) deterministic DP approach; (c) uncertainty‐informed approach based on CL masks at the optimal threshold $CL_{th}$. DP, deterministic prediction; ICC, intraclass correlation coefficient.

The analysis was first performed without including segmentation uncertainty in the workflow, using GT and DP masks to compute $ICC_{S}$ and either GT or DP masks separated to compute $ICC_{A}$ (Figure 2a). In this way, we can have a direct comparison with manual annotations and DL‐based contours without confidence correction. Subsequently, the analysis was conducted considering the masks ${\mathrm{CL}}_{th}$ at different confidence levels th for both ICC computations to evaluate the impact of incorporating segmentation confidence information (Figure 2b).

For each of the two analyses, features were classified into four classes based on $ICC_{S}$ and $ICC_{A}$ values, with a threshold of 0.8 for both, as this value was reported to be the most adopted criterion for evaluating radiomic stability.^42^ More specifically, they were classified as follows:

- Class 1, when $ICC_{S}\geq 0.8$ and $ICC_{A}\geq 0.8$, indicating both reproducibility and repeatability with respect to segmentation and scan–rescan variability, respectively;
- Class 2, when $ICC_{S}\geq 0.8$ and $ICC_{A}<0.8$, indicating only reproducibility with respect to segmentation variability;
- Class 3, when $ICC_{S}<0.8$ and $ICC_{A}\geq 0.8$, indicating only repeatability with respect to scan–rescan variability;
- Class 4, when $ICC_{S}<0.8$ and $ICC_{A}<0.8$, indicating absence of both reproducibility and repeatability with respect to segmentation and scan–rescan variability, respectively.

Moreover, for the analysis that integrates the evaluation of segmentation uncertainty considering the $CL_{th}$ masks, an optimal threshold $th_{opt}$ was defined as the threshold that minimizes the number of features classified into Class 4 (i.e., features that are neither reproducible with respect to segmentation variability nor repeatable with respect to scan–rescan variability). This choice was motivated by initially excluding unstable features while preserving those that demonstrated stability in at least one dimension. If this criterion was satisfied by multiple threshold values, $th_{opt}$ was the one that maximizes the number of features classified into Class 1 (i.e., features that are both reproducible across segmentations and repeatable across scan–rescan acquisitions).

Stability analysis was performed considering HC and CKD subjects separately, and performed both on the entire set of radiomic features and within individual feature categories (shape, first‐order, and texture matrices).

### 2.7 Classification analysis for CKD versus HC discrimination

To assess whether integrating segmentation uncertainty quantification into the radiomic workflow improves both feature stability and clinical relevance, we evaluated its impact on distinguishing between CKD and HC subjects. For this purpose, a classification model was developed for each segmentation method investigated in this study (GT, DP, MCD, TTA, ${\text{MCD}}_{\text{PLUS}}$, and ${\text{TTA}}_{\text{PLUS}}$).

Only radiomic features identified as the most reliable in the stability analysis were considered, because features with high intra‐ or inter‐observer variability, or high scan–rescan variability, are unlikely to be informative or generalizable, as suggested in the guidelines for radiomic model construction^43^ and observed in recent studies.^44^ Features with zero variance and those highly correlated with volume (correlation coefficient r>0.8 with *shape_MeshVolume*) were excluded. The minimum redundancy maximum relevance (MRMR) method, combined with bootstrapping (500 samples), was then applied to select the five most relevant features for each segmentation approach.

Logistic regression models were trained using the selected features of each segmentation approach, and evaluated using a leave‐one‐patient‐out cross‐validation strategy on the test set. Model performance was assessed by computing the receiver operating characteristic (ROC) curve, with the AUC used as the primary metric. Additionally, the DeLong test was performed to determine statistically significant differences between the GT and DP methods and each uncertainty‐based approach, with a significance level of 0.05.

## 3 RESULTS

### 3.1 Segmentation accuracy and uncertainty evaluation

Table 1 presents the obtained DSC_3D_ and KL divergence values for each stochastic network, for CKD and HC subjects separately.

**TABLE 1:** DSC_3D_ and KL divergence for each stochastic network, for CKD and HC subjects separately.

		MCD	TTA	${\text{MCD}}_{\text{PLUS}}$	${\text{TTA}}_{\text{PLUS}}$
**DSC_3D_**	CKD	0.91±0.02	0.90±0.02	0.91±0.02	0.91±0.02
	HC	0.94±0.01	0.93±0.02	0.92±0.02	0.93±0.02
**KL divergence**	CKD	0.11±0.03	0.79±0.19	0.91±0.21	1.42±0.29
	HC	0.12±0.04	1.07±0.22	0.93±0.28	1.51±0.26

Abbreviations: CKD, chronic kidney disease; DSC, Dice score coefficient; HC, healthy controls; MCD, Monte Carlo dropout; TTA, test‐time augmentation.

The accuracy of these methods was comparable to that achieved in the reference work,^24^ in which the authors reported a DSC_3D_ of 0.94±0.02 for HCs and of 0.92±0.01 for CKDs. All models achieved a slightly lower DSC_3D_ on CKD subjects.

Concerning the impact of the loss function, $L_{\text{DSC++}}$ slightly decreased model accuracy. However, this loss, which guaranteed improved model calibration, also ensured higher uncertainty for incorrectly classified pixels.

**FIGURE 3:**
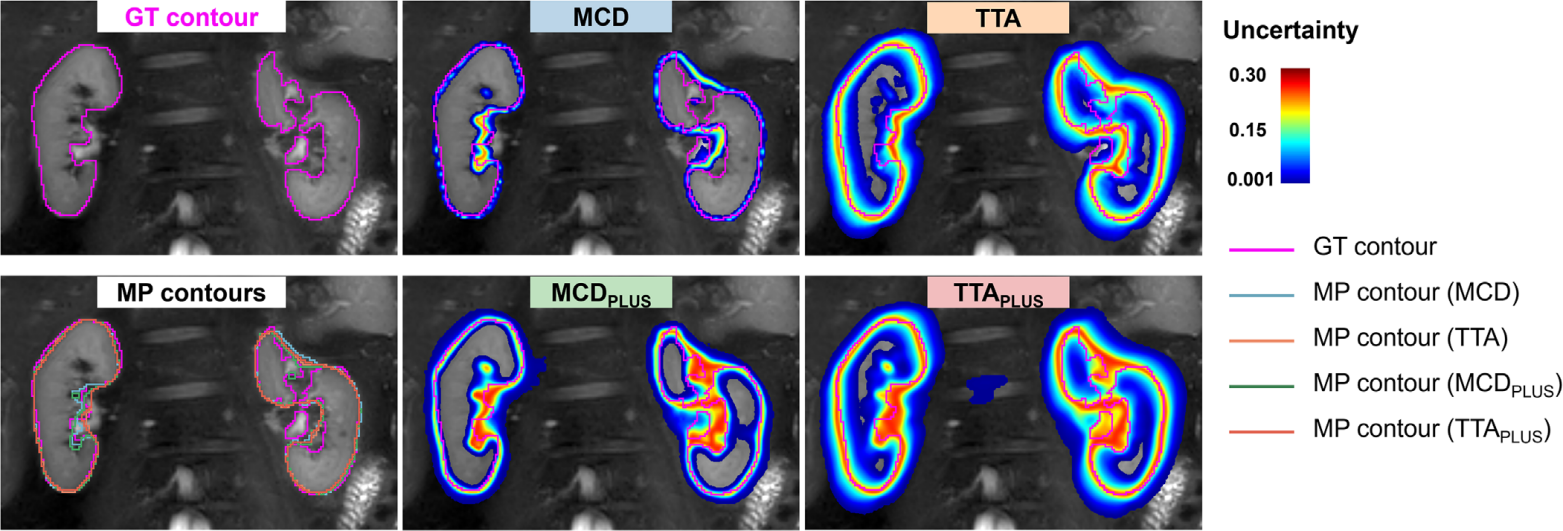
Kidney outlines obtained by the ground truth method and four stochastic models in a representative CKD patient. Top left: GT contour. Bottom left: MP contours obtained by the four methods. Middle and right: GT contour (pink line) and uncertainty map obtained for each method. Regions of the image with no uncertainty in the map correspond to areas where segmentation uncertainty is extremely low (below 0.001). CKD, chronic kidney disease; GT, ground truth; MP, mean prediction.

Such results can be also appreciated in Figure 3, which is related to a representative pathological case. This figure shows on the left the GT kidneys' contours and MP contours for the four models, which are very close to each other. Then, uncertainty maps are represented, together with the GT contour, for each method.

When training the models with $L_{\text{DSC}}$, uncertainty was very low and defined mainly along the kidney contour, as expected and confirmed by the low KL divergence found for the MCD and TTA models. When training was performed with $L_{\text{DSC++}}$, higher uncertainty also characterized more internal regions, especially those that were the most difficult to outline, since they were more unclear.

Regarding the comparison of the two proposed approaches for segmentation uncertainty estimation, TTA allowed for the enlargement of the zones of uncertainty with both loss functions. In the ${\text{TTA}}_{\text{PLUS}}$ model, however, uncertainty affected almost the entire structure of the kidney, thus becoming less informative.

### 3.2 Stability analysis

#### 3.2.1 Stability analysis without uncertainty information

Table 2 a details the results related to the stability analysis carried out without including the information about segmentation uncertainty.

**TABLE 2:** Stability analysis results obtained without accounting for segmentation uncertainty (a) and with segmentation uncertainty (b), using the optimal threshold ($th_{opt}$).

		Class 1	Class 2	Class 3	Class 4
		$ICC_{S}\geq 0.8$	$ICC_{S}\geq 0.8$	$ICC_{S}<0.8$	$ICC_{S}<0.8$
		$ICC_{A}\geq 0.8$	$ICC_{A}<0.8$	$ICC_{A}\geq 0.8$	$ICC_{A}<0.8$
**(a) Without segmentation uncertainty information**					
**GT**	CKD	19 (18.10%)	8 (7.62%)	5 (4.76%)	73 (69.52%)
	HC	13 (12.38%)	28 (26.67%)	11 (10.48%)	53 (50.48%)
**DP**	CKD	19 (18.10%)	8 (7.62%)	30 (28.57%)	48 (45.71%)
	HC	27 (25.71%)	14 (13.33%)	22 (20.95%)	42 (40.00%)
**(b) With segmentation uncertainty information (for** th = ${th}_{\text{opt}}$)					
**MCD**	CKD	21 (20.00%)	4 (3.81%)	43 (40.95%)	37 (35.24%)
	HC	42 (40.00%)	13 (12.38%)	22 (20.95%)	28 (26.67%)
**TTA**	CKD	20 (19.05%)	8 (7.62%)	41 (39.05%)	36 (34.29%)
	HC	29 (27.62%)	11 (10.48%)	30 (28.57%)	35 (33.33%)
${\text{MCD}}_{\text{PLUS}}$	CKD	20 (19.05%)	8 (7.62%)	50 (47.62%)	27 (25.71%)
	HC	14 (13.33%)	6 (5.71%)	56 (53.33%)	29 (27.62%)
${\text{TTA}}_{\text{PLUS}}$	CKD	18 (17.14%)	6 (5.71%)	38 (36.19%)	43 (40.95%)
	HC	22 (20.95%)	13 (12.38%)	34 (32.38%)	36 (34.29%)

*Note*: The table shows the number of features (with percentages) in each class for CKD and HC subjects separately.

Abbreviations: CKD, chronic kidney disease; DP, deterministic prediction; GT, ground truth; HC, healthy controls; ICC, intraclass correlation coefficient; MCD, Monte Carlo dropout; TTA, test‐time augmentation

When GT contours were used for this assessment, the majority of radiomic features were poorly stable with respect to both segmentation and scan–rescan variability, being in Class 4. This was true in both CKD and HC subjects.

However, a higher number of features were not affected by segmentation variability in HC subjects compared to CKD patients, as evidenced by the higher prevalence of reproducible features in Classes 1 and 2 (total number of 41 for HC vs. 27 for CKD). On the contrary, CKD and HC subjects presented the same number of repeatable features with respect to scan–rescan variability (sum of Classes 1 and 3, for a total of 24 features in both groups).

When DP masks were considered, the number of features in Class 4 decreased for both CKD and HC subjects (48 and 42, respectively), with an increased number of repeatable features in Classes 1 and 3 (49 in both groups).

#### 3.2.2 Stability analysis with uncertainty information

**FIGURE 4:**
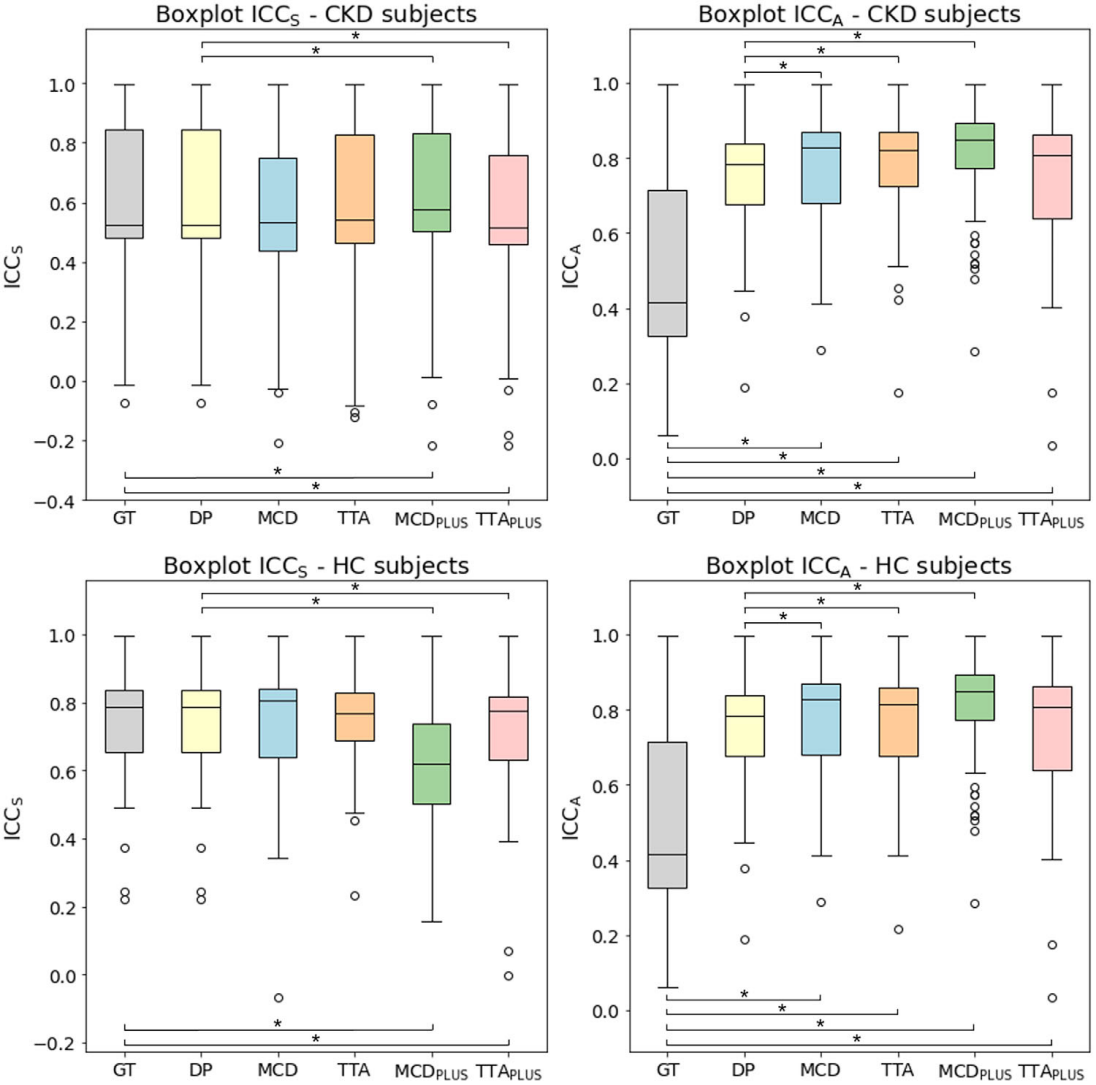
Boxplots of $ICC_{S}$ (on the left) and $ICC_{A}$ (on the right) values for CKD (top panel) and HC (bottom panel) subjects, separately for the GT and DP approaches and for each method integrating segmentation uncertainty estimation. Bars with asterisks indicate statistically significant results (*p*‐value < 0.05) between the GT/DP approach and each uncertainty‐based approach. CKD, chronic kidney disease; DP, deterministic prediction; DSC, Dice score coefficient; GT, ground truth; HC, healthy controls.

Figure 4 illustrates the distributions of $ICC_{S}$ and $ICC_{A}$ for each deterministic and segmentation uncertainty‐based approach. Results show that, for both CKD and HC subjects, the $ICC_{A}$ values obtained by all uncertainty‐based approaches are significantly higher compared to the GT approach. Similarly, all uncertainty‐based methods—except the ${\text{TTA}}_{\text{PLUS}}$—also show significantly higher $ICC_{A}$ values compared to the DP approach. Regarding $ICC_{S}$ values, instead, no clear trend is evident from the statistical analysis. Additional results were also presented in Table S1.

**FIGURE 5:**
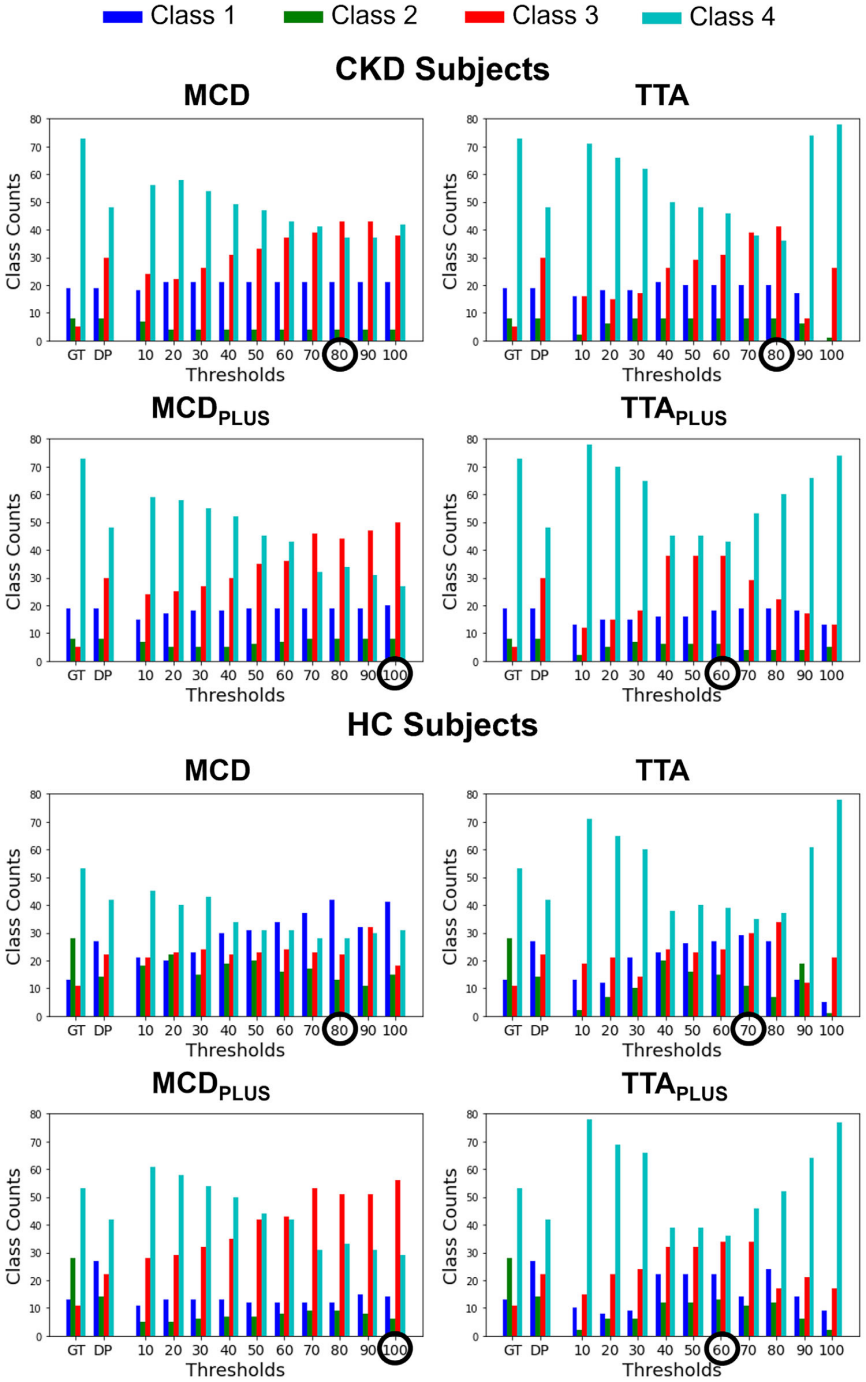
Results of the stability analysis both with and without accounting for segmentation uncertainty. The figure reports, for each method and in CKD patients (top) and HC subjects (bottom) separately, the number of features belonging to each class when not evaluating segmentation uncertainty (GT and DP) and based on the confidence level th. The optimal threshold for the confidence level $th_{opt}$ is indicated within the circles. CKD, chronic kidney disease; DP, deterministic prediction; GT, ground truth; HC, healthy controls.

Figure 5 illustrates the results of the radiomics stability analysis both with and without accounting for segmentation uncertainty. The figure reports, for each method and in CKD patients and HC subjects separately, the number of features belonging to each class when features were extracted from manually or deterministically defined ROIs, as well as the number of features in each class based on the confidence level th used to define the ROIs from which features were extracted.

**FIGURE 6:**
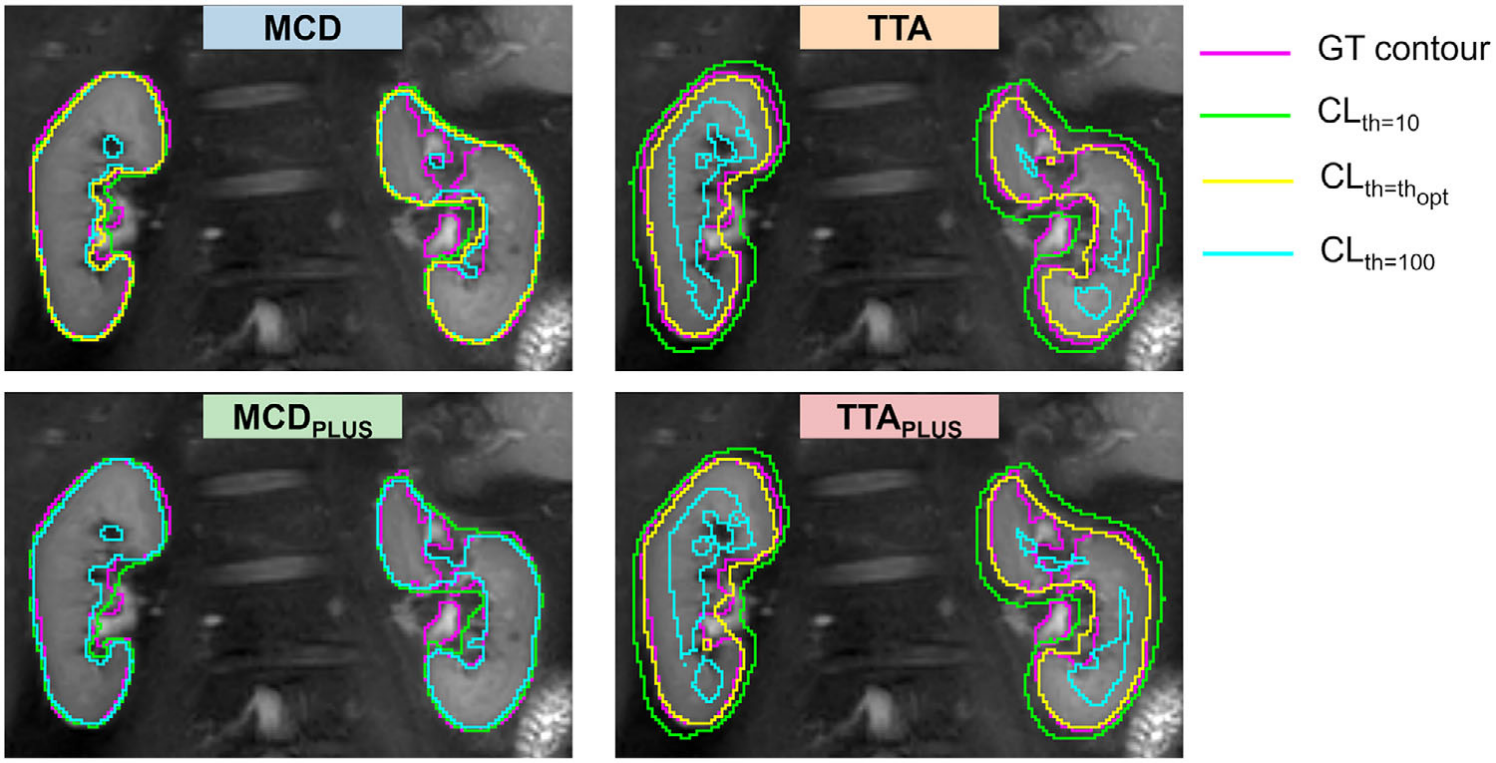
Kidney contours obtained in a representative CKD patient by the four stochastic models with different confidence levels th (10: green, optimal: yellow, 100: light blue) in comparison with the GT contours (pink). For ${\text{MCD}}_{\text{PLUS}}$, the optimal threshold is 100; thus, the yellow and light blue contours coincide. CKD, chronic kidney disease; GT, ground truth; MCD, Monte Carlo dropout.

All the methods, for both CKD patients and HC subjects, showed a common trend: compared to GT approach, the number of features in Class 3 increased with increasing segmentation confidence, while the number of features in Class 4 reduced accordingly. This trend held up to a certain confidence level th, which differed for each approach. For both HC subjects and CKD patients, $th_{opt}$ was 80 for MCD, 100 for ${\text{MCD}}_{\text{PLUS}}$, and 60 for ${\text{TTA}}_{\text{PLUS}}$. For TTA, $th_{opt}$ differed by subject group, being 80 for CKD patients and 70 for HC subjects. The lower $th_{opt}$ (60) found for TTA can be explained considering the example reported in Figure 6. By increasing the confidence level, the ${\text{TTA}}_{\text{PLUS}}$ kidney contour included only small internal regions of the kidneys, resulting in the decreased performance of the approach.

For both healthy and pathological cases, $th_{opt}$ identified for each method ensured an improvement in features' repeatability with respect to scan–rescan variability, compared to the GT and also the DP approach. Among the different methods, the MCD method implemented with $L_{\text{DSC++}}$ achieved the best performance, with 70 out of 105 features stable to scan–rescan variability, including 10 shape features, 11 first‐order features, and 49 texture features. This is clearly shown in Table 2 b and in Figure S3.

No improvement in reproducibility with respect to segmentation variability was observed for CKD subjects. The number of features in Class 2 remained approximately constant at different uncertainty levels. For HC subjects, on the other hand, improvements in reproducibility with respect to segmentation variability were achieved when using $th_{opt}$ with the MCD approach. A list of the features found stable in both CKD and HC subjects is provided in Table S2.

When radiomic feature categories (shape, firstorder, GLCM, GLRLM, GLSZM, GLDM, NGTDM) were considered separately, multiple $th_{opt}$ values that minimize features in Class 4 and maximize features in Class 1 were found, especially for categories including a small number of features. For most of the categories, $th_{opt}$ values were similar to those globally defined over all features. The complete list of $th_{opt}$ for each method, considering each radiomic feature category separately as well as the entire set, is reported in Table S3.

Looking at the stability results in the different radiomic categories, a noticeable enhancement in repeatability with respect to scan–rescan variability, particularly for first‐order and texture features, was found (see Table S3). Specifically, the percentage of first‐order features with $ICC_{A}\geq 0.8$ was of 33.33% for the GT approach, and increased to 66.67%, 72.22%, 61.11%, 61.11%, and 66.68% for DP, MCD, TTA, ${\text{MCD}}_{\text{PLUS}}$, and ${\text{TTA}}_{\text{PLUS}}$, respectively. Regarding the texture features, the percentage of repeatable features in relation to scan–rescan variability was, on average, 9.18% for the GT approach, increased to 38.44% for DP, and improved again to 53.68%, 48.98%, 68.91%, and 46.27% for MCD, TTA, ${\text{MCD}}_{\text{PLUS}}$ and ${\text{TTA}}_{\text{PLUS}}$, respectively. In contrast, a slight decrease in repeatability with respect to scan–rescan variability was observed for shape‐related features, with a more relevant reduction in the percentage of features with $ICC_{A}\geq 0.8$ for the ${\text{TTA}}_{\text{PLUS}}$ approach (from 92.86% for the GT approach to 57.14% for ${\text{TTA}}_{\text{PLUS}}$).

### 3.3 Classification analysis

Classification models were trained using the radiomic features identified as repeatable with respect to scan–rescan variability in the specific approach. This choice was made considering the results from the previous stability analysis, which highlighted that the major impact of introducing segmentation uncertainty in the radiomic workflow is the improved repeatability of the radiomic features to scan–rescan variability. Additionally, the features identified as repeatable with respect to scan–rescan variability were consistent for both CKD and HC subjects, thus allowing a larger set of features to be available to build classification models.

The five most significant features that were identified by the MRMR technique for the GT approach were associated with shape‐related parameters, while the introduction of DL‐based segmentation in the other methods enhanced variability in the selected features, which also included texture categories (see Table 3).

**TABLE 3:** List of the most relevant radiomic features selected by the MRMR technique with and without accounting for segmentation uncertainty.

Approach	Selected features using MRMR
GT	shape_Sphericity, shape_Elongation, shape_MajorAxisLength, shape_Flatness, shape_MeshVolume
DP	glszm_SizeZoneNonUniformity, gldm_DependenceNonUniformity, firstorder_Range, shape_Elongation, firstorder_Maximum
MCD	glszm_SizeZoneNonUniformity, gldm_DependenceNonUniformity, firstorder_Range, shape_Flatness, shape_Elongation
TTA	glszm_SizeZoneNonUniformity, shape_MeshVolume, shape_Sphericity, firstorder_Range, shape_Elongation
${\text{MCD}}_{\text{PLUS}}$	glszm_SizeZoneNonUniformity, firstorder_Range, shape_Flatness, firstorder_Maximum, ngtdm_Strength
${\text{TTA}}_{\text{PLUS}}$	glszm_SizeZoneNonUniformity, firstorder_Range, gldm_DependenceNonUniformity, shape_Flatness, firstorder_Maximum

Abbreviations: DP, deterministic prediction; DSC, Dice score coefficient; GT, ground truth; HC, healthy controls; MCD, Monte Carlo dropout; MRMR, minimum redundancy maximum relevance; TTA, test‐time augmentation.

**FIGURE 7:**
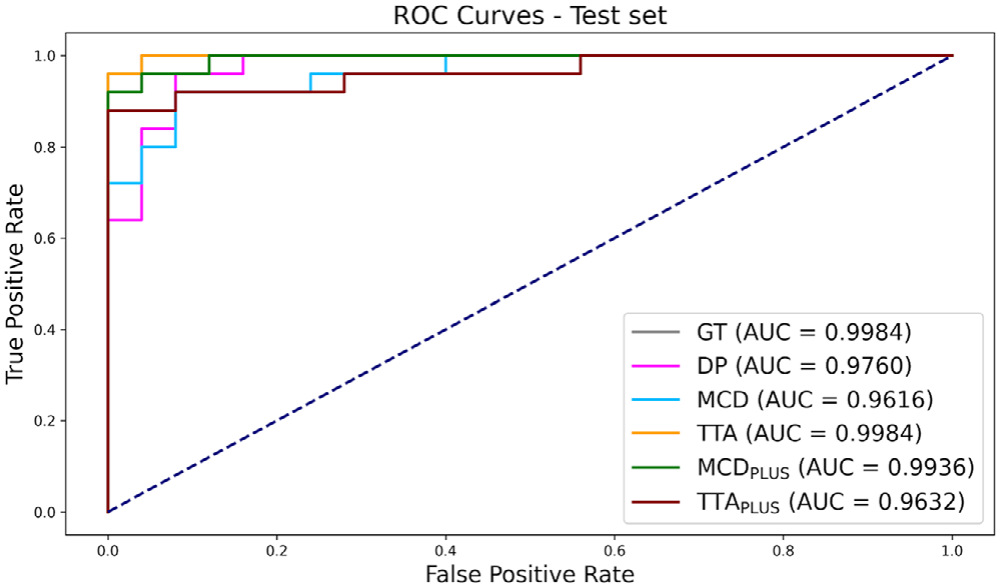
ROC curves for the GT and DP approaches, and for each uncertainty‐based segmentation approach. The AUC values for each method are also provided. The bisector of the figure is shown as a reference line indicating random performance. AUC, area under the curve; DP, deterministic prediction; GT, ground truth; ROC, receiver operating characteristic.

The assessment of classification performances through ROC curves is presented in Figure 7. The uncertainty‐based approaches showed a discriminatory power comparable to the GT approach, and slightly higher than DP model (AUC = 0.9616, 0.9984, 0.9936, and 0.9632 for MCD, TTA, ${\text{MCD}}_{\text{PLUS}}$, and ${\text{TTA}}_{\text{PLUS}}$, respectively, with respect to AUC = 0.9984 for GT and AUC = 0.976 for DP). No statistically significant differences were found.

## 4 DISCUSSION AND CONCLUSION

In this study, we aimed to investigate whether incorporating an uncertainty quantification step into the DL‐based segmentation process within the radiomic workflow could enhance feature stability with respect to segmentation and scan–rescan variability, and could increase the discriminatory power.

Our findings demonstrated that uncertainty‐informed ROI segmentation performed as part of the radiomic pipeline improved repeatability with respect to scan–rescan variability. The definition of an optimal confidence level for uncertainty allowed the number of repeatable features to increase with respect to scan–rescan variability compared to methods that did not incorporate uncertainty analysis. This improvement was consistent across both clinical scenarios (HC subjects and CKD patients) and for all methods investigated, with ${\text{MCD}}_{\text{PLUS}}$ showing a larger number of repeatable features. Additionally, the classification analysis revealed that the models based on uncertainty‐informed segmentation provided AUC values comparable to the reference GT approach, with higher performance for ${\text{MCD}}_{\text{PLUS}}$ and TTA, and that the relevant radiomic features selected by uncertainty‐informed segmentations included different texture categories, whereas the GT‐based approach was based on shape features only. In this sense, it is interesting to observe that the use of DL‐based segmentations allows the inclusion of intensity‐ and texture‐related features in the model for the discrimination of HC and CKD subjects, thus providing evidence that differences between the two groups are not only related to kidney volume and shape, but also to intensity patterns associated with structural tissue composition. The relatively straightforward classification task, in which shape‐related features alone provided strong discriminatory power, may have limited the ability to demonstrate the added value of incorporating confidence information. The relevance and potential benefit of uncertainty‐informed models should be further validated in more complex scenarios where texture‐based features play a more prominent role.

The improvement in repeatability with respect to scan–rescan variability observed when considering DL‐based masks can be attributed to the fact that automatic ROI segmentation itself already helps reduce intra‐ and interobserver variability.^16^ By focusing exclusively on the most certain segmentation regions, we further decreased variability by filtering out less reliable areas and concentrating on regions where the model output was more confident. This effect was particularly pronounced with the MCD implementation, as $th_{opt}$ (80 and 100 using $L_{\text{DSC}}$ and $L_{\text{DSC++}}$, respectively, for both HC and CKD subjects) significantly deviated from the median value, which approximated the network's output without including uncertainty information in the segmentation represented by the DP mask.

Looking at the radiomic features groups, texture and first‐order features benefited most from the introduction of uncertainty evaluation in ROI definition. For these, the increase in repeatability was consistent across all classes and methods tested, and may also justify the larger heterogeneity in the feature sets selected to build the classification models. In particular, GLCM features showed the most significant enhancement, suggesting that by focusing on regions with more confidently segmented boundaries, we can exclude high‐contrast areas that may vary across acquisitions, leading to more reliable feature extraction. Additionally, shape‐related features slightly decreased when accounting for uncertainty, with the ${\text{TTA}}_{\text{PLUS}}$ approach showing the least repeatable results. This finding suggests that the level of confidence in segmentation has a minor impact on the intrinsic properties of shape.

The approach proposed in this work could represent a valid strategy to mitigate scan–rescan related variabilities arising from patient‐specific factors, such as patient repositioning. This type of variability cannot be corrected using preprocessing or harmonization techniques and is typically addressed by discarding nonstable features. Our approach, instead, allows for the inclusion of a higher number of reliable features.

Although not investigated in this study, this technique could also be promising for addressing other acquisition‐related variabilities, such as those resulting from the use of different scanners (e.g., manufacturer, model, magnetic field strength) or from variations in acquisition protocols (e.g., MRI sequences, geometrical characteristics). The proposed uncertainty‐based approach may provide a beneficial alternative to traditional preprocessing methods, such as filtering, normalization, and resampling. Preprocessing techniques often involve interpolation, which can alter the original texture of an image, as noted in Scalco et al.,^45^ where the effect of normalization on radiomics was investigated. In contrast, our uncertainty‐based approach operates directly on the original ROIs, preserving the image's informational content. However, this aspect warrants further investigation.

Reproducibility with respect to segmentation variability, contrary to repeatability with respect to scan–rescan variability, showed improvement using the uncertainty‐based approach only in healthy subjects and remained unaffected in CKD patients. This discrepancy, along with the generally higher segmentation accuracy observed in healthy cases, consistent with the findings of Daniel et al.,^24^ can be attributed to the less complex and more consistent anatomical structures of healthy kidneys. In fact, healthy kidneys tend to have more consistent and uniform anatomical features, which makes segmentation more straightforward and less prone to variability. Consequently, uncertainty quantification methods can more effectively filter out unreliable regions, as demonstrated by the higher KL divergence found in HC subjects compared to CKD patients. In this way, uncertain pixels are more likely related to incorrect classification and, by excluding them from the segmentation, it is possible to obtain more stable features, thus improving reproducibility. In contrast, pathological kidneys exhibit significant variability and complexity due to pathological changes in size, shape, and internal structure, such as scarring, cyst formation, and uneven tissue density,^46^ which complicate accurate segmentation. These pathological variations introduce greater segmentation difficulty and variability, which uncertainty maps alone cannot fully mitigate. As a result, uncertain pixels may be related to both correct and incorrect classification, and thus, reproducibility improvements seen in healthy subjects are not observed in CKD patients. This suggests that while uncertainty quantification can mitigate challenges associated with scan–rescan variability, segmentation variability remains a considerable challenge, particularly in pathological cases.

The impact of the $L_{\text{DSC++}}$ on uncertainty quantification is significant, as it enhances the informativeness of the uncertainty maps, allowing the model to capture uncertainty not only near the kidney's contours but also in internal regions with complex tissue variations that contribute to increased segmentation difficulty. This observation is consistent with previous studies, such as the work by Yeung et al.,^28^ which demonstrated that training with the $L_{\text{DSC++}}$ improves model calibration by reducing overconfidence in incorrect predictions. In the same study, the authors also showed that in a retinal vessel segmentation task from fundus photographs, the $L_{\text{DSC++}}$ highlighted uncertainty in low‐confidence areas, particularly around the smaller, difficult‐to‐segment retinal vessels. Our results support these findings.

In our work, this improvement is quantitatively supported by the observed increase in KL divergence between the uncertainty distributions of correctly and incorrectly classified voxels. The higher KL divergence with $L_{\text{DSC++}}$ indicates that the uncertainty is significantly greater for misclassified voxels compared to correctly classified ones, thereby allowing the model to better represent regions of high uncertainty. This suggests that the $L_{\text{DSC++}}$ effectively improves the model's ability to identify and represent regions of higher uncertainty in medical image segmentation tasks.

The two approaches for uncertainty segmentation used in the current study (MCD and TTA) showed different behaviors. In particular, TTA showed the largest areas of uncertainty, with the highest values, when training with either the $L_{\text{DSC}}$ or the $L_{\text{DSC++}}$. Such results confirm that TTA is more conservative than MCD, which is instead more risk‐taking, since it fails to highlight uncertainty in the correspondence of some incorrect classified regions, as previously evidenced by Scalco et al.^30^ In our analysis, both the method used for uncertainty quantification and the loss function had a relevant impact on the choice of $th_{opt}$. MCD provided thinner regions than TTA, and thus the adoption of the $L_{\text{DSC++}}$ helped in removing the majority of incorrect pixels. On the other hand, the use of the TTA approach, estimating larger uncertain areas, combined with the $L_{\text{DSC++}}$ found the optimal region as the median segmentation, to avoid considering too small inner regions. Our findings suggest that $th_{opt}$ for uncertainty quantification should be adjusted according to the specific method applied, indicating the need for method‐specific tuning.

According to our study, none of the models consistently outperforms the others. Only a tendency of ${\text{MCD}}_{\text{PLUS}}$ and TTA to obtain a larger number of repeatable features and a higher AUC in the classification performance was observed. However, it can be suggested that models accounting for segmentation uncertainty, which demonstrate greater repeatability to scan–rescan variability and high discriminatory power, may be more suitable than deterministic approaches for longitudinal studies, in which such variability plays a key role.

These results highlight several important considerations. While the proposed approach shows promise, further examination is necessary to fully assess its robustness, especially to validate the choice of the optimal threshold in selecting the confidence level masks. Testing on additional datasets and more complex cases is essential for a comprehensive evaluation. This includes applications on other imaging modalities, such as other MRI sequences and CT scans, or on structures that present greater segmentation challenges, as well as on more challenging classification tasks, such as patient stratification. In this study, segmentation was evaluated on relatively simple structures with a high DSC_3D_ (above 0.9), but it is crucial to investigate the impact on more complex structures, such as renal tumors or cysts, where precise contouring is needed for boundaries that are difficult to define. In such cases, uncertainty could significantly expand across the mask, reducing the ROIs available for feature extraction when filtering out uncertain regions. Understanding whether this reduction meaningfully affects radiomics stability is an important direction for future research.

Additionally, in this work, only two methods for uncertainty quantification in DL‐based segmentation were tested. As a future development, it would be valuable to explore other uncertainty quantification approaches to assess the generalizability of our findings across different methods and determine which approach offers the best results in terms of robustness and accuracy. This would contribute to a more comprehensive understanding of which techniques are most effective for improving the stability of radiomic features.

In conclusion, integrating uncertainty quantification into DL‐based segmentation within the radiomics pipeline holds significant promise for addressing segmentation and scan–rescan variability challenges. The proposed uncertainty‐based approach enhances the reliability of radiomic analysis, maintaining at the same time a high discriminatory power, potentially facilitating the broader adoption of radiomics in clinical practice. This approach could lead to more informed and personalized clinical decisions, ultimately improving patient outcomes.

## CONFLICT OF INTEREST STATEMENT

The authors declare no conflicts of interest.

## Supporting information

Supporting Information
