# Supplementary material for: The impact of uncertainty estimation on radiomic segmentation reproducibility and scan–rescan repeatability in kidney MRI
Source: Med Phys. 2025 Jul 15;52(7):e17995. doi: 10.1002/mp.17995 (PMC12264328; doi:10.1002/mp.17995)
Supplement: Supplementary file 1 — Supporting Information [file MP-52-0-s001.pdf]

SUPPPLEMENTARY MATERIAL DESCRIPTION

Supplementary Material Figure S1:

An overview of the deterministic 2D U-Net architecture, assumed as reference in this work. It was implemented as in Daniel et al. [1].

Supplementary Material Figure S2:

An overview of the Bayesian 2D U-Net architecture, implemented for the MCD approach. Dropout layers are inserted and kept active during both training and testing phases.

Supplementary Material Figure S3:

Results of the reproducibility analysis both with and without accounting for segmentation uncertainty. The figure reports for each method and in CKD patients (top) and HC subjects (bottom) separately the number of features reproducible with respect to segmentation ($ICC_S \geq 0.8$) and acquisition variability ($ICC_A \geq 0.8$) when not evaluating segmentation uncertainty (GT and DT approaches) and based on the confidence level th. The optimal threshold for the confidence level th_{opt} is indicated within the circles.

Supplementary Material Table S1:

Supplementary Material Table S1 contains tables reporting the results of analyses conducted on individual radiomic features.

In table "ICC_OptimalThreshold", for each radiomic feature, ICC_s and ICC_A values are reported for CKD and HC subjects separately, for GT and DP approaches and for each stochastic method in correspondence of the optimal confidence level.

In tables "ICC_MCD_AllThresholds", "ICC_TTA_AllThresholds", "ICC_MCDPLUS_AllThresholds" and "ICC_TTAPLUS_AllThresholds", for each radiomic feature, ICC_S and ICC_A values are reported in correspondence of all the confidence level thresholds, for CKD and HC subjects separately and for MCD, TTA, MCD_{PLUS} and TTA_{PLUS} methods respectively.

Supplementary Material Table S2:

Table "StableFeatures(Class1)" presents, for each implemented method, the list of features identified as stable and thus classified as Class 1 — that is, features that are both reproducible with respect to segmentation variability and repeatable with respect to scan-rescan variability, for both CKD and HC subjects.

Table "RepeatableFeatures(Class1+Class3)" presents, for each implemented method, the list of features identified as repeatable with respect to scan-rescan variability (Class 1 and Class 3 features), for both CKD and HC subjects. These features were used as the starting set for the implementation of the classification models distinguishing CKD from HC subjects.

Supplementary Material Table S3:

Supplementary Material Table S3 contains tables reporting the results of analyses conducted on categories of radiomic features (shape, firstorder, GLCM, GLRLM, GLSZM, GLDM, NGTDM).

Table "List_OptimalThresholds_Group" reports the optimal thresholds (th_{opt}) for each uncertainty-based approach, separately for CKD and HC subjects, across each feature category as well as for the overall feature set.

The tables 'MCD_GroupFeaturesAnalysis', 'TTA_GroupFeaturesAnalysis', 'MCDPLUS_GroupFeaturesAnalysis', and 'TTAPLUS_GroupFeaturesAnalysis' report, for each feature category and each class, the percentage of features with respect to the total number of features in that category, separately for CKD and HC subjects, for GT and DP approaches and for all the confidence level thresholds for MCD, TTA, MCD_{PLUS} and TTA_{PLUS} approaches respectively.

In table "Summary1_GroupFeaturesAnalisys", percentages of features within each features' category with ICC_S and ICC_A values above the threshold of 0.8 are reported for CKD and HC subjects separately, for the GT and DP approaches and for each stochastic method in correspondence of the optimal confidence level th_{opt}.

In table "Summary2_GroupFeaturesAnalysis", the mean of ICC_S and ICC_A values of features within each category are reported for CKD and HC subjects separately, for GT and DP approaches and for each stochastic method in correspondence of the optimal confidence level.

[1] Daniel, Alexander J., et al. "Automated renal segmentation in healthy and chronic kidney disease subjects using a convolutional neural network." *Magnetic resonance in medicine* 86.2 (2021): 1125-1136.
